# Investigating the accessibility factors that influence antenatal care services utilisation in Mangwe district, Zimbabwe

**DOI:** 10.4102/phcfm.v9i1.1337

**Published:** 2017-06-29

**Authors:** Leoba Nyathi, Augustine K. Tugli, Takalani G. Tshitangano, Molyn Mpofu

**Affiliations:** 1 Department of Public Health, University of Venda, South Africa; 2 Department of Curriculum studies, University of Venda, South Africa

## Abstract

**Background:**

Maternal and infant mortality remains a huge public health problem in developing countries. One of the strategies to minimise the risks of both maternal and infant mortality is access to and utilisation of antenatal care (ANC) services.

**Aim:**

This study aimed to investigate the accessibility factors that influence the use of ANC services in Mangwe district.

**Methods:**

A qualitative approach using explorative design was adopted to target women who have babies under 1 year of age. The study was conducted in Mangwe district, Matabeleland South province, Zimbabwe. Data were collected through semi-structured interviews and observations. Data saturation was reached after 15 women who were conveniently sampled were interviewed. Field notes were analysed thematically using Tech’s steps. Lincoln and Guba’s criteria ensured trustworthiness of the study findings.

**Results:**

Accessibility factors such as lack of transport, high transport costs and long distances to health care facilities, health care workers’ attitudes, type and quality of services as well as delays in receiving care influence women’s utilisation of ANC services in Mangwe district, Zimbabwe.

**Conclusion:**

The study concluded that women were still facing problems of unavailability of nearby clinics; therefore, it was recommended that the government should avail resources for women to use.

**Recommendations:**

Mangwe District Health Department should provide mobile clinics rendering ANC services in distant rural areas.

## Introduction

Maternal and infant mortality remains a huge public health problem in developing countries. One of the strategies to minimise the risks of both maternal and infant mortality is access to and utilisation of antenatal care (ANC) services endorsed by WHO that recommends a minimum of four focused antenatal check-ups.^1^ Proximity is a key factor for access; adequate transport, good roads and communication networks are essential to reach the poor and physically isolated crowds.^2^ Access to health services includes gaining entry into the health care system, finding a health care provider with whom the patient can communicate and trust, and accessing a health care location where needed services are provided.^2,3^ The benefits of access to health care include overall physical, social and mental wellbeing; prevention of diseases and disability, and detection and treatment of health conditions; prevention of avoidable death; increasing life expectancy and improving quality of life.^4,5^

Knowledge on the provision of services, physical and financial accessibility of services, and knowledge on how best to utilise services and cultural norms of treatment are important factors to be considered.^1^ Limited access to and utilisation of health care increase morbidity and mortality rates;^6^ therefore, it is paramount to provide action-based approaches that are effective in rural areas which will include developed infrastructure such as roads, public transportation and primary health care facilities.^3^

World Health Statistics in a recent analytical review of ANC coverage showed that between 2006 and 2013, ANC was indirectly correlated with maternal mortality ratio (MMR) worldwide.^7^ Therefore, this shows that countries with low ANC coverage will have a high MMR.^7,8^ Despite the United Nations Sustainable Development Goal 3, which is to ensure healthy lives and promote well-being for all at all ages by 2030, four out of every five deaths of children under age 5 occur in Asian and sub-Saharan regions including Zimbabwe. In addition, MMR, that is, the proportion of mothers who do not survive childbirth compared with those who do, in developing regions is still 14 times higher than that in the developed regions. Furthermore, only half of women in developing regions receive the recommended amount of health care they need.^8,9,10^ Several studies associate the above facts with barriers to health care service such as lack of availability, high cost and lack of insurance coverage,^11,12,13^ challenges of access to important health services like effective ambulance and other emergency transportation as a result of poor road infrastructure.^8^ This study aims to verify these claims by investigating the accessibility factors of ANC services utilisation in Mangwe district, Zimbabwe.

## Research methodology and design

### Study design

The study adopted a qualitative approach using exploratory design.

### Setting

This study was conducted in Mabunga village, Mangwe district, Zimbabwe. Mangwe district is located in Matabeleland South province, in south-western Zimbabwe, close to the international border with Botswana. It is a sub-district of Bulilimamangwe district. Its main town, Plumtree, is located about 100 km by road, south-west of Bulawayo, the nearest large city.

### Study population and sampling strategy

Permission to access records of women who attend ANC at Marula clinic was granted by the district administrator (DA), and the DA also allowed the investigator to proceed to Mabunga village. The headman of Mabunga village allowed the researcher to move around the village searching for participants with the help of the village health worker. The target population was sufficiently representative of different groups with regard to age, parity, socioeconomic status, marital status and level of education.

The population included mothers who had babies of age under 12 months at the time of the study. A sample of 15 women were chosen based on the criterion of data saturation, where the researcher noted that they were receiving the same information from the participants. Purposive sampling was used in this study where the researcher searched for participants who met the selection criteria. These women were then approached depending on their availability at the time of study. Appointments were made with the participants.

### Data collection

The first interview gave the researcher an opportunity to test if the research questions were clear. Questions were then adjusted in response to the pre-test findings. Data were collected through semi-structured interviews with individual participants. The purpose of the study was explained to the participants, and each participant signed a consent form. The time for interviews was set to suit the availability of each participant. Data were collected within eight days. Pseudo names were used so as to maintain confidentiality. The participants were told of the main reason for the use of pseudo names. All the participants were asked open-ended questions.

The interviews were conducted in Ndebele by the researcher as all participants were Ndebele speaking. Each interview lasted between 45 min and 1 h. The interviews were audio taped and transcribed verbatim within 24 h of the interview. They were then translated into English before being analysed. Field notes were written immediately after each interview to describe the physical setting and the activities which occurred during each interview. The collected data were analysed using the thematic analytical approach.

### Data analysis

The collected data were analysed using the thematic analytical approach. All data were transcribed and then translated from Ndebele to English. Reliability of transcriptions was achieved through reading them while listening to the recordings. The researcher was familiarised with the depth and breadth of the content. A thorough analysis of the entire data set was performed once more before beginning to code, as the ideas, and the identification of possible patterns was shaped in the process of reading through. The data were initially coded and collated, and there was a long list of different codes the researcher would have identified across the data set. This phase, which re-focuses the analysis at the broader level of themes, rather than codes, involved sorting the different codes into potential themes, and collating all the relevant coded data extracts within the identified themes. The researcher devised a set of themes and refined them. During this phase, some themes collapsed into each other whereas other themes needed to be broken down into separate entities. The researcher had a satisfactory thematic map of data. The themes were defined and further refined, and they were not too diverse or complex.

The Lincoln and Guba model of trustworthiness was adopted which includes credibility, confirmability, dependability and transferability;^13^ these are described below:

#### Credibility

To ensure credibility, the researcher actively participated in the collection of data. Prolonged engagement, taking down of field notes and observation of the participants’ responses were performed. The researcher gained a deeper understanding of the topic as well as specific aspects of the participant’s perceptions through the interviews. Findings were shown to the participants to allow them to confirm if they represent their opinions accurately and to ensure credibility of the tape recorders and field notes used in the data collection.

#### Dependability

In this study, dependability was achieved by describing the research findings, interpretations and recommendations, using an auditable trail so as to corroborate that the investigation was supported by data and was internally coherent. A voice recorder was used to increase reliability of all interviews.

#### Confirmability

Confirmability in this study was ensured by the use of an independent coder who went through the transcriptions with the researcher and together they reached a consensus on the themes found. This was reflected by the voice of the participants and not the researcher’s perceptions. This was supported by integration of an audit procedure where the researcher described all the research processes, explained and justified what they intend to do and made presentations on the reasons for undertaking decisions.

#### Transferability

The researcher provided a complete description of the research methodology findings and verbatim quotes from individual interviews to ensure applicability of the study to other contexts. The researcher requested someone with research experience to randomly read selected transcripts and to identify major categories, so that readers may have a clear picture of the findings.

#### Ethical considerations

Approval and ethical clearance were obtained from University of Venda Higher Degree and Research Ethics committees (SHS/15/PH/15/0707). A written informed consent was obtained from all participants prior to data collection.

## Results

Five themes emerged during data analysis, namely lack of transport, high transport costs for ANC visits, long distance to the health facility, health care workers’ attitudes towards ANC women, type and quality of care rendered and delays in receiving care.

### Theme 1: Lack of transport

Transport was raised as a major issue that played a key role in ANC services access. Most women in the study mentioned that they faced difficulties accessing transport as there was only one common transport that was used, which did not come every day. According to participants, shortage of transport also resulted in women using Kezi rural hospital which is situated in Matobo district, 40 km distance in a different direction from Mangwe. The road to Marula clinic in Mangwe district was bad in such a way that taxis could not use it. Thus, it was convenient for them to use Kezi hospital, although the distance to Marula was shorter than that to Kezi hospital. The following is participants’ exact words:

‘I rarely see transport going to Marula, the road is too bad. There is only one transport, a bus, which comes only once a day. The Kezi people welcomed us with open arms as they know our situation of unavailability of a nearer clinic and transport problems.’ (P7, female, 36 years)

The researcher observed that indeed the roads were bad and hence available transportation to that area was a serious problem.

### Theme 2: High transport cost for antenatal care visits

Women expressed concern about high transport costs. They mentioned that because they were not working, it was difficult for them to pay for the transport fee as it was expensive. Some needed to be accompanied by a family member and it meant double costs. The following is what they said:

‘… But the transport costs are terrible. I have to pay $8.00 to and fro which is too much for my husband and I as we are both not employed, especially when I need to be accompanied. We only depend on piece jobs.’ (P2, female, 36 years)

### Theme 3: Long distance to the health facility

Most women stated distance to the clinic as their reason for not attending ANC services regularly. The distance to the hospital is more than 40 km and with the type of transport they use, it meant that they would spend the whole day at the hospital and come back at night. Participants said:

‘Kezi hospital is very far from our place. What happens is that we wake up early in the morning around 4 a.m. and come back around 11p.m. because the transport will be going to Bulawayo in the morning and will pick us up on its way back.’ (P7, female, 36 years)

The researcher also noted that the hospital was too far from the village leading to some women even giving birth at home especially in an emergency.

### Theme 4: Lack of proper roads

Women cited lack of bridge as their reason for not using ANC services regularly. Thus, during the rainy season they are affected as they become immobile because of the lack of transport. Participants said:

‘During the heavy rains, the river gets full therefore the transport cannot cross as there is no bridge. Which means that if I have an appointment around that time, it will be impossible to honour it.’ (P9, female, 30 years)

The researcher noted that Mangwe and Matobo district are separated by a big river called Shashane; therefore, during the rainy season it is impossible for people to cross to the other district as there is no bridge to allow them.

### Theme 5: Health care workers’ attitude and approach towards antenatal care women

Most women did not seem to have any problem with the health care workers’ attitudes towards patients. Most of them reported that the nurses were treating them very well, giving them useful information. However, two participants claimed that the reason why they attended ANC once is because of the attitudes of nurses. They said:

‘The reason why I did not continue to go there is because those nurses harass us, saying we come late to the clinic, forgetting that we stay too far away.’ (P4, female, 24 years)

‘The nurse just comes with a serious face and tells you to remove your clothes. The next thing they just stick their fingers inside you and they don’t say anything. They just do their things quickly so as to get rid of us. They are not friendly or approachable.’ (P9, female, 30 years)

### Theme 6: Type and quality of services provided

Most participants reported that they did not receive advice about birth preparedness and signs of pregnancy complications during their pregnancy check-up visits at the health centre. According to these women, nurses take much time writing things on paper. In this study, most participants did not seem to know anything about the ultrasound check and medication they were given.

‘Do they have time to teach us? Even the pills they give you, you are just given without explanation. I only got educated once and we were in a group. I feel they should also do individual education so that we get to express ourselves easily.’ (P10, female, 33 years)

### Theme 7: Delays in receiving care

Some women complained that they were often made to wait long periods, sometimes dismissed because nurses would claim that it was too late for ANC. Women were more concerned about spending the whole day at the hospital which means that they had to buy food while waiting for their turn to be served. They said:

‘When we arrive early there, the long queues are just unbearable, such that one can spend the whole day on the queues. Sometimes you are told to go back home unattended, nurses will tell you that it is late for ANC.’ (P4, female, 24 years)

## Discussions

Results in this study revealed that transport was a major barrier to accessing ANC services. Similar findings were discovered in Kenya, Ghana and Malawi where vehicles providing public transport were scarce and women mainly walked to the clinic and some travelled on their husband’s bicycle.^15,16^ Scarcity of transport also resulted in women using Kezi Rural Hospital, which is situated in Matobo district, quite a long distance away. This is because the road to Marula Clinic in Mangwe district was bad such that taxis could not use it; therefore, it was convenient for them to use the hospital although the distance to Marula was shorter than that to Kezi Hospital. Studies have shown that uncomfortable transport, poor road conditions and difficulties in crossing big rivers were also barriers to the utilisation of ANC services.^11,17^ An example is that of Tanzania which revealed that in Mkuranga district there was a serious shortage of transport which was contributed to by poor road conditions and this discouraged public transport owners to operate in that area.^18^

Another important factor raised was the expensive transport costs. This made the women not attend ANC as recommended, as they will be trying to avoid the costs that are associated with visiting health facilities. Findings from other studies^17^ show that although ANC services are available, women initiate late because of lack of money to continue attendance. Furthermore, in Nigeria cost was discovered as an important factor contributing to non-access to maternal health care facilities.^19^ All the women who earned more than N20 000 ($148) per month received ANC, had more frequent antenatal visits, were more likely to use orthodox facilities for delivery and none of them delivered at home.^20^

A study in Canada revealed that in the islands, a woman had to pack for herself and her family for a long journey of up to seven weeks to seek better health care services, leading to strains in the family.^21^ In Nigeria, it was also discovered that many pregnant women found it distressing to walk long distances or take two or more buses in an effort to avail ANC on appointment days.^22^ This is consistent with other studies where an association between distance to a health facility and ANC use was found in that an increase in distance to the nearest health facility led to fewer antenatal visits but did not influence the timing of the first antenatal visit.^1,23^

Some women in the study seemed to be satisfied with the attitude and approach they were given by health care workers. This is supported by a study investigating pregnant African women’s perceptions on maternity services in Australia, where women described the health care workers as sensitive, accommodating and tolerant to them as foreigners.^24^ However, some women reported that nurses did not give them sufficient information on the medicines they were giving them, and they also do certain procedures without explaining to the patient, which makes the woman uncomfortable. In contrast, South African women reported that nurses yelled and scolded patients and some of them preferred to help those who are wealthy rather than the poor.^25^ In Zimbabwe, respondents described midwives as hostile and abusive, which might affect the attitudes to and utilisation of ANC.^26^

Some women showed dissatisfaction with the quality of services rendered to them at the hospital. One important component of ANC is to offer information and counsel women about birth preparedness and promote the importance of institutional delivery; therefore, health workers are expected to provide information on danger signs during pregnancy and other benefits of institutional delivery based on the principles of focused ANC.^26,27,28^

## Conclusion

This article concludes that several access factors such as lack of transport, high transport costs, long distances to health care facilities, health care workers’ attitude types and quality of service as well as delays in receiving care influence women’s utilisation of ANC services in Mangwe district, Zimbabwe.

## Recommendations

It is therefore recommended that Mangwe District Health Department should provide mobile clinics rendering ANC services in distant rural areas. It should strengthen maternal health programmes and must ensure that they are efficient, friendly, focused and accessible. Funds must be made available for the provision of mobile clinic services rendering ANC for easy accessibility. Government and non-governmental organisations must support community initiatives to build a clinic for the Mabunga community and neighbouring villages that can benefit from it.
